# 

**Published:** 1825-04

**Authors:** 


### MONTHLY CATALOGUE OF MEDICAL BOOKS.

Illustrations of the Arteries connected with Aneurism and Surgical Operations. These Plates are intended to explain the relative Position of the Arteries in respect to the surrounding parts and the Organs to be met with in such Operations, both externally and internally to their sheaths. No. II. containing Plates 2, 3, and 4, of a complete series. By G. D. Dermott, m.r.c.s.—London, 1825.

☞ As Works of this kind do not admit of review, we take this opportunity of expressing our approbation of the manner in which these plates are executed The object is sufficiently explained in the title-page; and we may merely mention that the present fasciculus contains three views, representing the different stages of the operation for taking up the upper part of the axillary artery, or the lower part of the subclavian below the clavicle. The first plate represents a superficial dissection of the axilla; the second, a deeper dissection of the same parts; and in the third, the parts are still more fully exposed. These plates are highly coloured, and accompanied by a copious letter-press description.—We must add, that the price is extremely moderate, being only 9*s*.—a sum considerably less than is usually charged for engravings executed in a similar style.

Part Second of a Series of Elementary Lectures on the Veterinary Art; wherein the Anatomy, Physiology, and Pathology of the Horse, are essayed on the general Principles of Medical Science. By WILLIAM PERCIVALL, Member of the Royal College of Surgeons in London; Licentiate of the Society of Apothecaries; and late Veterinary Surgeon of the Royal Artillery.—London, 1824; pp. 559.

An Estimate of the true Value of Vaccination as a Security against Small-Pox. By T. M. GREENHOW, Member of the Royal College of Surgeons in London; Surgeon to the Lying-in Hospital, to the Charity for poor Married Women lying-in at their own Houses, and to the Infirmary for Diseases of the Eye, Newcastle. —London, 1825; pp. 75.

Dissertatio Chemico-Physiologica de Sanguine ejusque Mutationibus: quam Annuente Summo Numine, ex Auctoritate reverendi admodum viri, D. GEORGII BAIRD, SS. T. P. Academiæ Edinburgenæ Præfecti, necnon Amplissimi Senatus Academici Consensu, et Nobilissimæ Facultatis Medicæ decreto; pro Gradu Doctoris, summisque in Medicina Honoribus ac Privilegiis Rite et Legitime Consequendis, Eruditorum examini Subjicit CAROLUS J. B. WILLIAMS, Anglus.—Edinburgi, MDCCCXXIV.; pp. 82.

A short Treatise on Operative Surgery, describing the principal Operations as they are practised in England and France; designed for the use of Students in operating on the dead Body. By CHARLES AVERILL, Member of the Royal College of Surgeons, of the Medico-Chirurgical Society, and Surgeon to the Cheltenham Dispensary and Casualty Hospital, &c. &c. The Second Edition, considerably enlarged, and with the addition of Drawings.—London, 1825; pp. 250.

---

### NOTICE TO CORRESPONDENTS.

Dr. HARRISON'S *Paper was too late for the present Number.*

*We hope to be able to insert* Mr. SHAW'S "*Remarks on Wounds received in post-mortem Examinations*" *in our next.*

Mr. WHITE'S *and* Mr. WATKINSON'S *Communications have been received.*

*We have been much amused with the satire on* Dr. K.'s *style; nevertheless we do not mean to publish it. He is an old Contributor to this Journal; and, though he sometimes indulges in a* copia verborum, *we have been unwilling to decline publishing his Communications. The evil, however, has recently been complained of by too many, not to convince us of the necessity of reforming this matter.* Verbum sat.

*We must decline inserting the Paper of* Mr. M. *on the very grounds he requested us to do it,—viz. that we are desirous to "act the part of fair and impartial Journalists." A fair criticism of the opinions of any Correspondent, we never object to, but the present is merely personal.*

*We cannot give insertion to the letter from* "A GENERAL PRACTITIONER."

*We must likewise request* Mr. T. *to excuse us for declining to insert his Paper: however absurd the doctrines which he ridicules, we do not think his remarks, as they stand at present, calculated to produce any good effect.*

Mr. E's *Case is of too frequent occurrence to warrant its publication.*

*We have received the letter from* Dr. M'S. *on removing Fluids from the Stomach by the "Gum-elastic Syphon." He will find that sufficient attention has already been bestowed upon this subject in this and other Journals.—(N.B. All these Communications have been returned to the Publisher, and may be had by proper application.)*

